# Biodegradation of Polyester Polyurethane by *Embarria clematidis*

**DOI:** 10.3389/fmicb.2022.874842

**Published:** 2022-06-14

**Authors:** Sarunpron Khruengsai, Teerapong Sripahco, Patcharee Pripdeevech

**Affiliations:** ^1^School of Science, Mae Fah Luang University, Chiang Rai, Thailand; ^2^Center of Chemical Innovation for Sustainability (CIS), Mae Fah Luang University, Chiang Rai, Thailand

**Keywords:** polyester polyurethane, biodegradation, *Embarria clematidis*, Fourier-transform infrared spectroscopy, gas chromatography-mass spectrometry, esterase

## Abstract

Polyester urethanes (PUR) are widely used in industries and have led to a worldwide plastic waste problem. Thus, novel solutions for PUR degradation are required to reduce environmental pollution. This work investigates the PUR biodegradation efficiency of 33 fungal species using a polyester-polyurethane colloid branded Impranil DLN (Impranil) compared to *Aspergillus niger*, which served as the positive control. The biodegradation is evaluated based on its ability to clear Impranil in media. Eleven fungi can clear Impranil in both solid- and liquid-medium assays. The highest degradation was attributed to *Embarria clematidis* cultured with Impranil as a carbon source. The degradation was confirmed by the Sturm test, Fourier-transform infrared (FTIR) spectroscopy, and gas chromatography-mass spectrometry (GC-MS). From the Sturm test, CO_2_ at a concentration of 0.85 g/L was found in *E. clematidis* cultured with 150 mL of Impranil solution after a 2-week incubation period while the CO_2_ at a concentration of 0.53 g/L was detected from *A. niger* in the same conditions. The biodegradation was further confirmed by evaluating the clearance percentage of supernatant of *E. clematidis* and *A. niger* culturing with Impranil from the Sturm test. The clearance percentage of *E. clematidis* and *A. niger* supernatant was 88.84 and 48.97%, respectively. Moreover, the degradation of soft segment and breakdown of ester linkages were observed, as evidenced by the decrease of the carbonyl (1,715 cm^–1^) and N-H stretching (1,340 cm^–1^ and 1,020 cm^–1^) FTIR spectral peaks, respectively. GC-MS detected 3Z-heptenol, 5Z-octenol, 2E,4E-hexadienol acetate, and 3E,6Z-nonadienol as degradation products from the *E. clematidis* culture supernatant. This fungus was screened for its ability to produce extracellular esterase, protease, and urease enzymes. Extracellular esterase, very low urease, and no protease activities were detected in the culture supernatant of *E. clematidis* in the presence of Impranil. *E. clematidis* can degrade Impranil partially *via* hydrolysis of ester linkages by cell-bound esterases at a considerable rate without any prior treatment. This fungus not only degraded Impranil but also mineralized them into CO_2_ and H_2_O. *E. clematidis* can be applied in the process of biochemical depolymerization of PUR for the pure monomers recycling.

## Introduction

Plastic has tremendously used due to its excellent physical properties such as versatility, malleability, chemical, and physical resistance, and ease of production compared to other materials. Polyester polyurethane (PUR) is a major plastic material that is widely used in many industrial applications to produce foams, elastomers, coatings, and adhesives (Howard, 2002). The estimated production of PUR is approximately 8 million tons annually (Matsumura et al., 2006). It is mainly produced *via* the nucleophilic reaction from two monomers (a diol and a highly toxic and reactive diisocyanate) and is derived from the toxic substance phosgene in the presence of some additives. Reprocessing of PUR generates toxic residues, such as aldehydes, ammonia, cyanide, isocyanates, nitrogen oxides, and vinyl chloride, which are hazardous to humans, disrupt ecological processes, and contribute to environmental pollution (Cregut et al., 2013). PUR is also physically stable, making it resistant to biological degradation (Yang et al., 2001). Moreover, the large amount of produced PUR materials has resulted in a massive increase in plastic waste (Miskolczi et al., 2004). Hence, effective methods for the degradation of plastic wastes are needed to solve the problems posed by PUR waste accumulation.

Recently, various bacteria and fungi have been demonstrated to degrade PUR materials. Fungi in some genera have been reported to degrade PUR, including *Alternaria*, *Aspergillus*, *Phoma*, *Penicillium*, *Plectosphaerella*, *Geomyces*, *Nectria*, and *Neonectria* (Cosgrove et al., 2007; Schmidt et al., 2017). In addition, *Aspergillus niger*, *Aspergillus flavus*, *Aspergillus vesicolor*, *Penicillium funicutosum*, *Aureobasidium pullulaus*, some species of *Trichoderma* sp., and *Chyaetomium glubosum* also can degrade PUR reported by Darby and Kaplan (1968). Some bacterial species, including a few species of *Corynebacterium* genus, *Enterobacter agglomerans*, and *Bacillus subtilis* have also been reported to efficiently degrade PUR samples (Rowe and Howard, 2002; Mahajan and Gupta, 2015; Schmidt et al., 2017). However, few organisms have been demonstrated to degrade PUR as the sole carbon source (Shah et al., 2008; Russell et al., 2011).

Several reports have indicated that the active enzymes involved in microbial degradation of PUR are esterases, lipases, proteases, and ureases, suggesting the degradation of the PUR substrate by the cleavage of the ester bond (Tokiwa and Calabia, 2004; Shah et al., 2008; Kale et al., 2015; Danso et al., 2019; Ru et al., 2020). These enzymes were isolated from various microorganisms such as *Pseudomonas fluorescens*, *Comamonas acidovorans*, *Pseudomonas chlororaphis*, *Pseusomonas aeruginosa*, *Acinetobacter gerneri*, *Bacillus subtilis* MZA-75, *Comamonas acidovorans* TB-35, *Aspergillus terreus*, and *Chaetomium globosum* (Allen et al., 1999; Yang et al., 2001; Shah et al., 2008). Although both fungi and bacteria have been reported to degrade PUR, the actual mechanism of the biodegradation process remains unknown. The ability of these microorganisms to degrade PUR offers the possibility of being able to degrade other complex polymers as well (Russell et al., 2011). A massive number of microorganisms could have the degradation ability to colonize the surface of PUR (Glaser, 2019). Therefore, investigating other PUR-degrading microorganisms and their enzymatic activities is essential for efficient PUR waste management (Liu et al., 2021).

This study presents the Impranil biodegradation potential by various fungi. The study also demonstrates their ability to degrade Impranil on a culture plate, in a liquid medium, and with Impranil as the sole carbon source. Analysis of its CO_2_ production, enzymatic activities, and degradation products was also performed.

## Materials and Methods

### Fungal Strains

Thirty-four fungal species were selected for this study (Table 1). The fungus *A. niger* was obtained from the Thailand Institute of Scientific and Technological Research, Bangkok, Thailand while the other strains were obtained from the culture collection of the Institute of Excellence in Fungal Research, Mae Fah Luang University, Thailand.

**TABLE 1:** List of fungi for screening of PUR degradation.

No.	MFLUCC code	Species	Genbank no.
1	MFLUCC 14-0259	*Pseudophaeosphaeria rubi*	KX765299
2	MFLUCC 14-0525	*Ophiosimulans tanaceti*	KU738891
3	MFLUCC 14-0561	*Murilentithecium clematidis*	KM408758
4	MFLUCC 14-0582	*Roussoella scabrispora*	KY026583
5	MFLUCC 14-0651	*Populacrescentia foricesenensis*	MG520925
6	MFLUCC 14-0652	*Embarria clematidis*	KT306593
7	MFLUCC 14-0958	*Sclerostagonospora lathyri*	MF398878
8	MFLUCC 15-0031	*Pseudomassariosphaeria bromicola*	NG059595
9	MFLUCC 15-0078	*Nodulosphaeria multiseptata*	KY496728
10	MFLUCC 15-0177	*Vagicola chlamydospora*	KU163654
11	MFLUCC 15-0381	*Halobyssothecium obiones*	MH376744
12	MFLUCC 15-0491	*Paraepicoccum amazonense*	KU900295
13	MFLUCC 15-0949	*Poaceascoma halophila*	MF615399
14	MFLUCC 16-0619	*Pestalotiopsis microspora*	KU900295
15	MFLUCC 17-0066	*Muyocopron heveae*	MH986828
16	MFLUCC 17-0075	*Muyocopron dipterocarpi*	MH986834
17	MFLUCC 17-0555	*Colletotrichum fructicola*	MG646969
18	MFLUCC 17-0571	*Colletotrichum pandanicola*	MG646931
19	MFLUCC 17-0582	*Alternaria burnsii*	MG646987
20	MFLUCC 17-0759	*Setoseptoria arundelensis*	MG828962
21	MFLUCC 17-0887	*Cytospora centravillosa*	MF190123
22	MFLUCC 18-0091	*Diaporthe italiana*	MH853690
23	MFLUCC 18-0093	*Coniella vitis*	MH569466
24	MFLUCC 18-0518	*Septomelanconiella thailandica* *	MH727706
25	MFLUCC 18-0675	*Murilentithecium lonicerae*	MK214370
26	MFLUCC 18-0677	*Phragmocamarosporium hederae*	MK214369
27	MFLUCC 18-0682	*Keissleriella caraganae*	MK214368
28	MFLUCC 18-0701	*Neomollisia gelatinosa*	MK591960
29	MFLUCC 18-0787	*Thyrostroma jaczewskii*	MK765857
30	MFLUCC 18-1018	*Tamsiniella labiosa*	MK034865
31	MFLUCC 18-1586	*Diaporthe rumicicola*	MH84623
32	MFLUCC 18-1593	*Epicoccum pseudokeratinophilum*	MH827002
33	MFLUCC 18-1595	*Stagonosporopsis citrulli*	MH827024
34		*Aspergillus niger* ATCC 10254	

*Species found only in Thailand.

### Screening of Impranil-Degrading Activity

All fungi were screened for their Impranil-degrading activity on solid-medium assay (Russell et al., 2011). Impranil, an anionic aliphatic aqueous PUR dispersion (Bayer MaterialScience, New Jersey, United States), was used to prepare the polymer solution. A solid medium was prepared following a protocol by Russell et al. (2011), which involves mixing 19 mM NaH_2_PO_4_, 33.5 mM K_2_HPO_4_, 7.6 mM (NH_4_)_2_SO_4_, 2.5 mM Na citrate, 250 μM MgSO_4_, 19 μM thiamine, 0.05% Casamino acids, 147 μM FeCl_3_⋅6H_2_O, 14 μM ZnCl_2_⋅4H_2_O, 12 μM CoCl_2_⋅6H_2_O, 12 μM Na_2_MoO_4_⋅2H_2_O, 10 μM CaCl_2_⋅2H_2_O, 11 μM CuCl_2_, 12 μM MnCl_2_, 12 μM H_3_BO_3_, 1.8 mM HCl, and 15 g of agar in 1,000 mL deionized water. Impranil (10 mL) was added to the medium after autoclaving to prevent deformation. A plug of fungi (5 mm diameter), which was cultured on potato dextrose agar (PDA) for 1 week, was placed at the center of solid medium plates. All plates were further kept at room temperature for 2 weeks. The plates containing the agar plug of *A. niger* were used as the positive control. The Impranil-degrading activity of the fungi was evaluated by inspecting for halos of clearance and colony diameter after 2 weeks of incubation.

### Analysis of Clearance

#### Solid-Medium Assay

The fungi that showed Impranil-degrading activity were further assayed for their clearance *via* the solid-medium screening assay, according to the method of Russell et al. (2011). The solid medium (20 mL) was placed in sterile tubes. A plug of fungi (5 mm diameter) cultured on PDA for 1 week was placed on top of each test tube before 2 weeks of incubation at room temperature. The fungus *A. niger* was used as a positive control for Impranil degradation. After 2 weeks of incubation, Impranil degradation was evaluated *via* the clearance of the medium, which changed from opaque to translucent. The clearance depth was measured from the top of the medium to the lowest point of the visible clearance.

#### Liquid-Medium Assay

The fungi that showed Impranil-degrading activity was also assayed for their clearance *via* the liquid-medium screening assay, according to a modified method described by Russell et al. (2011). The liquid medium was prepared using the same composition found in the solid medium but without agar. The liquid medium (150 mL) was added to a sterile culture flask and inoculated with five plugs of fungus grown on PDA for 1 week. All culture flasks were further incubated at room temperature for 2 weeks. At the end of incubation, the fungal mycelium was separated from the culture supernatant using vacuum filtration. The obtained liquid supernatant was homogenized by vigorous shaking and the absorbance of the supernatant liquid was measured using a UV-visible PerkinElmer spectrophotometer at a wavelength of 600 nm. The sterile deionized water was used as a blank. Series dilutions of liquid medium prepared in sterile water were used to construct a standard curve for converting absorbance to percent clearance. The fungus *A. niger* was used as a positive control.

### Sole Carbon Source Assay

The fungi that showed Impranil-degrading activity were tested for their ability to use Impranil as the sole carbon source, following a modified method described by Russell et al. (2011). To ensure that there was no other carbon source, Impranil was only used as the sole carbon source for metabolism and fungal growth. The fungus *A. niger* was tested as a positive control. Each fungus was cultured in a potato dextrose broth (PDB) for 1 week. Stock cultures were homogenized by vigorous shaking and further centrifuged at 3,000 rpm for 5 min. The supernatant was removed and the fungal pellets were washed three times with sterile distilled water to confirm the complete removal of residual PDB. The fungal pellet was then added to 150 mL of liquid medium, which was prepared similar to the liquid-medium assay without agar and salts. The visual clearance of the opaque culture supernatant was determined every 2 days for 2 weeks by measuring the optical density at 600 nm.

### CO_2_ Production Assay

The amount of CO_2_ released by *E. clematidis* in the presence and absence of Impranil as the sole carbon source after a 2-week inoculation was investigated. The modified Sturm test was used in this experiment. Sterile was first flowed through 1 M KOH solution to remove atmospheric CO_2_ before analysis. The amount of dissolved CO_2_ in the medium broth was determined every 2 days for 2 weeks by titration method (Ameen et al., 2016). The medium broth was filtered and 25 mL of filtrate was mixed with 0.05 mL of 0.1 N Na_2_S_2_O_3_ and 2 drops of methyl orange. The solution was titrated with 0.02 M NaOH solution until the solution turned yellow. After that, two drops of phenolphthalein were added to the solution, and titration was continued until the solution turned pink. The volume of the used titrant was recorded. The CO_2_ amount was calculated using the following equation: [A × B × 50 × 1,000]/V, where A = volume of NaOH in mL, B = normality of NaOH, and V = volume of sample in mL. The fungus *A. niger* was used as a positive control.

### Fourier-Transform Infrared Spectroscopy Analysis

Degradation by *E. clematidis* in the presence of Impranil as the sole carbon source in a liquid medium was also characterized by FTIR (Álvarez-Barragán et al., 2016). After the 2-week incubation, the mycelium was removed by filtration filter paper and 1 mL of each filtrate was placed in sample cell before its spectra was collected using FTIR (Lumos, Bruker) with 64 scans at a resolution of 4 cm^–1^. All spectra were presented within the wavenumber range of 500–4,000 cm^–1^. Deionized water spectrum was used for the background spectrum. The results were compared to the spectra obtained from the *A. niger* culture in the presence of Impranil as the sole carbon source after 2-week inoculation and the liquid medium without fungal culture.

### GC-MS Analysis

After 2-week incubation, 20 mL of filtered culture supernatants of *E. clematidis* and *A. niger* in the presence of Impranil as the sole carbon source were partitioned with 20 mL of dichloromethane (Sigma Chemical Co., St. Louis) using a separatory funnel. The volatile organic compounds was analyzed using the GC-MS system (Agilent Technologies, Santa Clara, CA, United States). The capillary column is used as an HP-5 ms column (30 m × 0.25 mm i.d., 0.25 μm film thickness) (Agilent Technologies, Santa Clara, CA, United States). The oven temperature was initially held at 60°C and then increased at a rate of 3°C/min to a final temperature of 220°C. The injector temperature was set to 250°C. Helium was used as a carrier gas with a flow rate of 1 mL/min. Electron impact mass spectra were collected at 70 eV over the range of m/z 30–300. The ion source and analyzer temperatures were set to 250 and 230°C, respectively. Identification of compounds was done by comparing their mass spectra with those found in the NIST14 Mass Spectral Library.

### Enzymatic Activity Assay

The enzymatic activity of esterase, lipase, and urease was measured using spectrophotometry. These enzymes were measured every 2 days from cultures of *E. clematidis* in the presence of Impranil as the sole carbon source following the protocol of Álvarez-Barragán et al. (2016). The filtrates were centrifuged at 10,000 × g for 10 min and the cell pellet was removed from the supernatant. The obtained supernatants were further filtered through 0.45 nm-pore size Millipore membranes, obtaining a solution with a total volume of 3 mL. Next, three dialysis rounds were sequentially carried out against 50 mM potassium phosphate buffer (pH 7.0) for 2 h (in 1,000 mL of buffer), 24 h (in 2,000 mL of buffer), and 2 h (in 1,000 mL of buffer). For 0–7 days of incubation, 1 μg of protein was used, whereas, for 8–14 days of incubation, 4 μg of protein was used for the enzymatic assays. Esterase activity was determined by the hydrolysis of p-nitrophenyl acetate (Sigma-Aldrich, United Kingdom). The reaction was prepared by mixing 50 mM potassium phosphate (pH 7.0), 100 mL of enzyme extract, and 5 mM p-NPA in a final volume of 1 mL, following a protocol introduced by Oceguera-Cervantes et al. (2007). Protease activity was determined by casein hydrolysis, following the modified method of Oceguera-Cervantes et al. (2007). The reaction solution contained 100 mM potassium phosphate (pH 7.0), 50 mL of enzyme extract, and 0.5% casein in a final volume of 1 before incubation at 37°C for 24 h. Urease activity was determined based on the amount of ammonia from a phenol hypochlorite assay, following a protocol by Oceguera-Cervantes et al. (2007). The reaction solution contained 15 mM potassium phosphate (pH 7.0), 100 mL of enzyme extract, and 3.8 mM urea in a final volume of 1.3 mL. The solutions were determined spectrophotometrically at 405, 280, and 636 nm for esterase, lipase, and urease activity assay, respectively. The results were compared to those obtained from the liquid medium without fungal culture and the *A. niger* in the presence and absence of Impranil.

### Statistical Analysis

All experiments were performed in triplicates. Data from each experiment were presented in terms of the mean ± standard deviation. Data were subjected to Analysis of Variance (ANOVA), followed by *post hoc* multiple pairwise comparisons using Duncan’s multiple range tests. The statistical analyses were conducted using the SPSS 20.0 software (IBM Corp.; 2011, NY, United States).

## Results

### Screening of Impranil-Degrading Fungi

**FIGURE 1:**
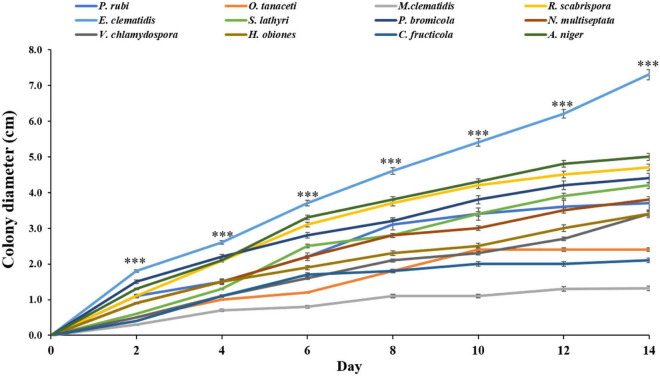
Colony diameter of active fungi on solid medium plates after 2-week inoculation. Sign ^***^ above the points indicates a significant difference (*p* < 0.001) (ANOVA, followed by Dunnett’s test).

Thirty-three fungal species were tested on their ability to degrade Impranil as compared to *A. niger*. After 2 weeks of incubation, the visible halo of clearance around the colony was evaluated and recorded. It was found that halos were detected in 12 fungal species, including *P. rubi*, *O. tanaceti*, *M. clematidis*, *R. scabrispora*, *E. clematidis*, *S. lathyri*, and *P. bromicola*, *N. multiseptata*, *V. chlamydospore*, *H. obiones*, *C. fructicola*, and *A. niger*. The colony diameter of these active fungi was also measured (Figure 1). *E. clematidis* exhibited the biggest colony diameter (7.3 cm). Some fungi, including *A. niger*, *V. chlamydospore*, *H. obiones*, *C. fructicola*, *N. multiseptata*, *S. lathyri*, *P. bromicola*, and *R. scabrispora*, exhibited a moderate colony diameter (3.4–4.7 cm), while *M. clematidis*, *P. rubi*, and *O. tanaceti* exhibited a small colony diameter of less than 3.0 cm.

### Analysis of Impranil Clearance

**FIGURE 2:**
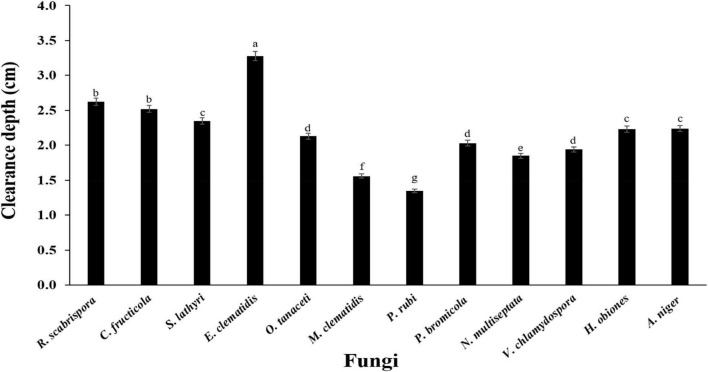
Clearance depth of solid medium of active fungi after 2-week inoculation. Different letters indicate significant differences (*p* < 0.05) (ANOVA, followed by Duncan’s multiple range test).

**FIGURE 3:**
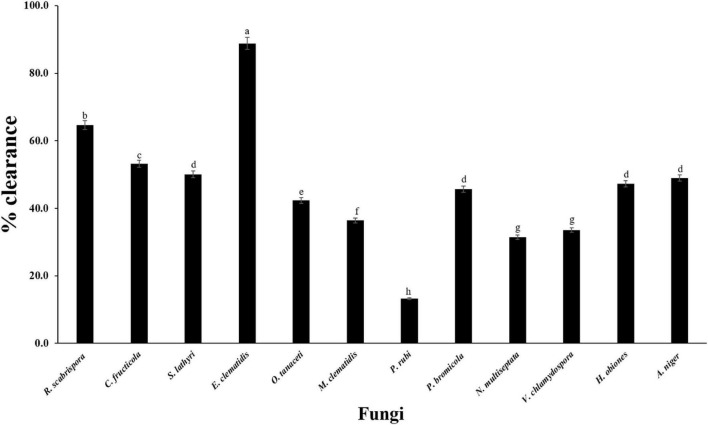
Clearance percentage of culture supernantant of active fungi after 2-week inoculation. Different letters indicate significant differences (*p* < 0.05) (ANOVA, followed by Duncan’s multiple range test).

The active fungi were tested in Impranil clearance assays using solid- and liquid-medium. In the solid-medium assay, each fungal plug was inoculated in test tubes with the solid medium and the clearance depth was measured after 2 weeks of incubation (Figure 2). All of the 12 active fungi visibly cleared the solid medium. Four fungi (*S. lathyri*, *C. fructicola*, *R. scabrispora*, and *E. clematidis)* cleared the medium more efficiently than the positive control fungus *A. niger* (2.24 cm). The most efficient fungi for Impranil degradation was *E. clematidis* with an average clearance depth of 3.28 cm. The Impranil clearance percentage of the active fungi was also measured in a liquid-medium assay (Figure 3). The liquid cultures were cleared to the point of visual transparency at the top of the liquid culture, extending down the length of the flask. The relative order of the liquid clearance assay was similar to those observed for the solid-medium assay. The *E. clematidis* was the most active fungus in the liquid-medium assay, followed by *R. scabrispora*, *C. fructicola*, and *S. lathyri* with a clearance rate of 88.84, 64.68, 53.19, and 50.05%, respectively. These fungi cleared the liquid culture better than *A. niger*, the positive fungal control, after 2 weeks of incubation.

### Sole Carbon Source Assay

**FIGURE 4:**
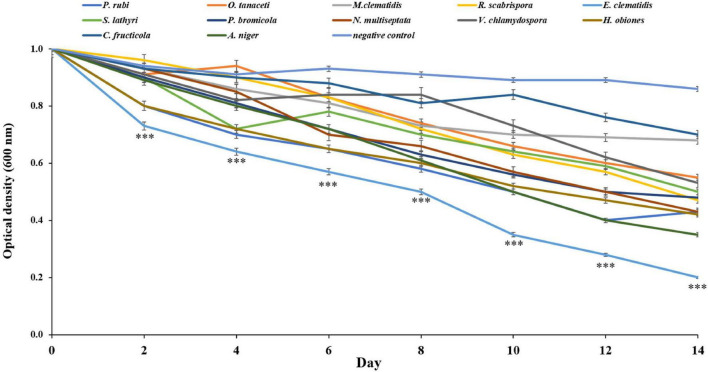
Optical density of culture supernatant of active fungi after 2-week inoculation. ^***^Above the points indicates a significant difference (*p* < 0.001) (ANOVA, followed by Dunnett’s test).

All active fungi were tested for their ability to degrade Impranil in liquid culture using Impranil as the sole carbon source. If fungus grows in the liquid medium, it means that Impranil can be used as the sole carbon source for its metabolism and growth. The tested fungi from the solid- and liquid-medium assays demonstrated Impranil degradation activity even under this minimal medium condition. To compare the degradation rates of Impranil, the active fungi were grown for 2 weeks. The optical density of the culture supernatant of active fungi after a 2-week inoculation period is depicted in Figure 4. The cultures were visually transparent by the end of the 2 weeks. *E. clematidis*, with an optical density value of 0.21, has the highest clearance rate among the active fungi including the control *A. niger*, which showed an optical density value of 0.35. The lowest clearance rate was found in *C. fructicola* with an optical density value of 0.71.

### CO_2_ Production

**FIGURE 5:**
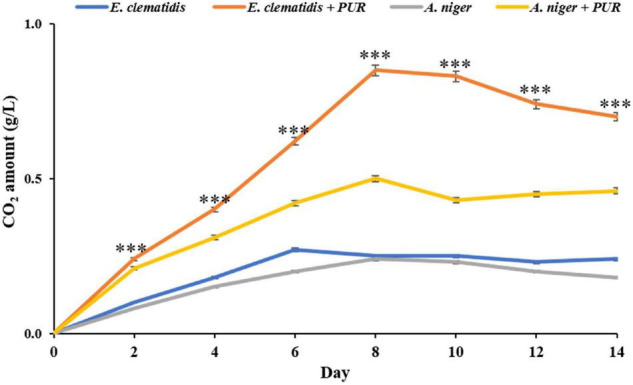
CO_2_ amount released by *E. clematidis* and *A. niger* in the presence and absence of Impranil as the sole carbon source after 2-week inoculation. ^***^Above the points indicates a significant difference (*p* < 0.001) (ANOVA, followed by Dunnett’s test).

The amounts of CO_2_ released by *E. clematidis* and *A. niger* in the presence and absence of Impranil after a 2-week incubation are shown in Figure 5. *E. clematidis* in the presence of Impranil demonstrated the highest CO_2_ amount among the tested treatments, with the highest amount detected on day 8 of the incubation. The CO_2_ concentration from the other treatments ranged from 0.00 to 0.85 g/L. *A. niger* in the presence of Impranil also demonstrated a high amount of CO_2_, ranging from 0.00 to 0.53 g/L, with the highest CO_2_ amount detected on day 8 as well. Similar CO_2_ amounts were detected in *E. clematidis* and *A. niger* in the absence of Impranil, ranging from 0.00 to 0.27 g/L and 0.00 to 0.24 g/L, respectively, after a 2-week incubation.

### Fourier-Transform Infrared Spectroscopy Analysis

**FIGURE 6:**
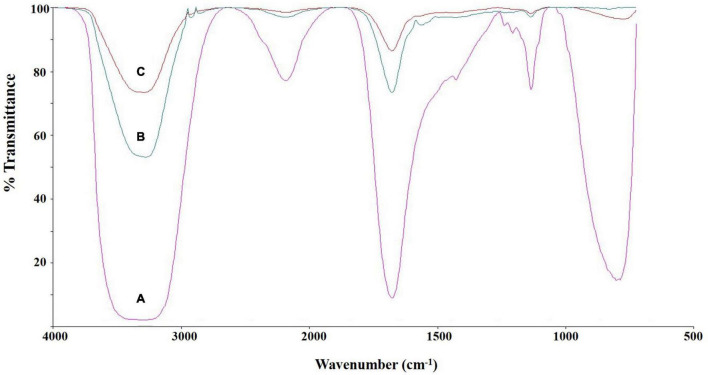
FTIR spectra of culture supernatant of *E. clematidis* **(C)**, and *A. niger* **(B)** in the presence of Impranil as sole carbon source, and Impranil liquid medium without culturing of fungi **(A)** after 2-week inoculation.

The chemical structure of culture broth during the Impranil degradation by *E. clematidis* was investigated using FTIR spectroscopy (Figure 6). The liquid medium without fungi displayed a large absorption peak at 1,715 cm^–1^, corresponding to the carbonyl (C=O) stretch of the ester linkage in the Impranil (Figure 6A). In addition, the low intensities of the C-N-H bending (1,340 cm^–1^) and stretching (1,020 cm^–1^) modes of the urethane group were also observed in the liquid medium without fungi. However, a significant decrease in the relative intensities of the peaks at 1,715 cm^–1^, 1,340 cm^–1^, and 1,020 cm^–1^ was detected in the visually transparent culture broths of *E. clematidis* (Figure 6C) and *A. niger* (Figure 6B) in the presence of Impranil as sole carbon sources. In addition, a strong peak broadening was observed at 3,370–3,135 cm^–1^ for the liquid medium without fungi, while a medium peak broadening at the same spectral window was observed for *E. clematidis* and *A. niger* in the presence of Impranil.

### GC-MS Analysis

**FIGURE 7:**
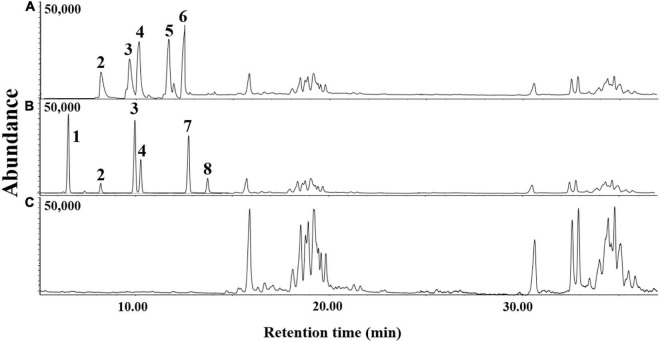
GC-MS chromatograms of dichloromethane extracts of *A. niger* **(A)** and *E. clematidis* **(B)** culture supernatant in the presence of Impranil as the sole carbon source, and Impranil liquid medium without culturing of fungi **(C)** after 2-week inoculation.

The volatile organic compounds of the culture supernatant of *E. clematidis* and *A. niger* in the presence of Impranil after a 2-week incubation period is shown in Figure 7 and Table 2, respectively. Eight chemical compounds were detected among both fungal cultures in presence of Impranil after 2-week incubation (Table 2). The major compounds in the *E. clematidis* culture supernatant in the presence of Impranil were 3Z-heptenol, 5Z-octenol, 2E,4E-hexadienol acetate, and 3E,6Z-nonadienol. Meanwhile, the compounds 5Z-octenol, 2E,4E-hexadienol acetate, 3Z-hexenyl isobutanoate, and phenyl-tert-butanol were detected as the major compounds in the *A. niger* culture supernatant in the presence of Impranil.

**TABLE 2:** Volatile organic compounds of dichloromethane extracts *E. clematidis* and *A. niger* culture supernatants in the presence of Impranil as sole carbon source after 2-week inoculation.

No.	Compound	*A. niger*	*E. clematidis*
1	3Z-heptenol		×
2	6-methyl-5-hepten-2-ol	×	×
3	5Z-octenol	×	×
4	2E,4E-hexadienol acetate	×	×
5	3Z-hexenyl isobutanoate	×	
6	phenyl-tert-butanol	×	
7	3E,6Z-non adienol		×
8	2E-non-enol		×

*×, presence.*

### Enzymatic Activity Assay

**FIGURE 8:**
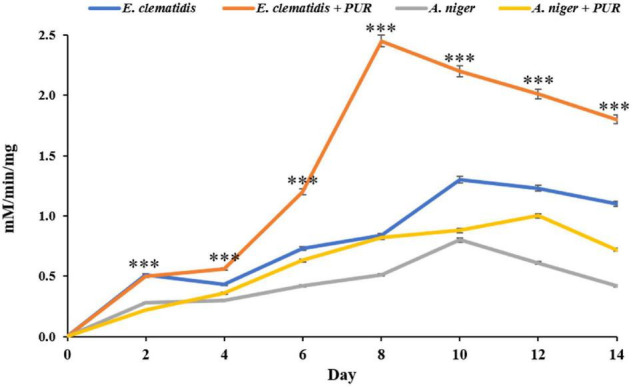
Esterase activity of *E. clematidis* and *A. niger* in the presence and absence of Impranil as the sole carbon source after 2-week inoculation. ^***^Above the points indicates a significant difference (*p* < 0.001) (ANOVA, followed by Dunnett’s test).

Protease activity was not found in supernatant extracts of *E. clematidis* and *A. niger* in the presence of Impranil after a 2-week incubation, and very low urease activity was determined among all treatments, based on the amount of NH_3_, ranging from 0.14 to 0.21 μM/min/μg. However, a high amount of esterase enzyme was detected in the supernatant extracts of *E. clematidis* and *A. niger*, as shown in Figure 8. Low esterase activity (< 1.0 mM/min/mg) was detected in cell supernatants of *A. niger* in the absence of Impranil after a 2-week incubation. The highest enzymatic activity was detected in the supernatant of the *E. clematidis* fungal culture broth in the presence of Impranil. A gradual increase in enzymatic activity in the *E. clematidis* cell in the presence of Impranil was found on days 0–8 (0.00–2.45 mM/min/mg), which then gradually decreased to 1.81 mM/min/mg after 2 weeks of incubation. The enzymatic activity of *E. clematidis* cell in the absence of Impranil increased from 0.52 to 1.31 mM/min/mg from day 0 to day 10 but then decreased to 1.14 mM/min/mg after day 14. In addition, the highest esterase activity of *A. niger* in the presence of Impranil was found on day 12 (0.88 mM/min/mg), followed by 0.72 mM/min/mg on day 14.

## Discussion

All Impranil-degrading fungi belong to the phylum Ascomycota. Several fungal species in this phylum have been reported to have PUR degradation ability, such as *Pestalotiopsis microspore* (Russell et al., 2011), *Cladosporium herbarum* (Álvarez-Barragán et al., 2016), *Cladosporium cladosporioides* (Álvarez-Barragán et al., 2016), *Penicillium griseofulvum* (Brunner et al., 2018), *Alicycliphilus* spp. (Oceguera-Cervantes et al., 2007), and *Xepiculopsis graminea* (Álvarez-Barragán et al., 2016). Although many fungi have already been reported as PUR degraders, most of the species that are described in this work, as well as their ability to degrade Impranil, have been rarely reported. Impranil was found to employ as a representative of PUR in searching for PUR-degrading microorganisms (Russell et al., 2011; Nakkabi et al., 2015; Hung et al., 2016; Magnin et al., 2019) due to their various useful attributes resulting it ideal for biodegradation assays such as its commercial availability, application as a coating material, stability to pH 4–8, tolerance to high temperature up to 80°C, and rapid rate of dissolution compared to other polyurethanes. However, the formulation of Impranil is proprietary and its colloidal state has ultimately made Impranil a precarious choice for polyurethane-degrading microorganisms (Biffinger et al., 2015). The active fungi showed different Impranil degradation abilities, probably due to differences in the physiology, biochemistry, and genetics among the fungal species (Brunner et al., 2018). The fungus *E. clematidis* showed the highest Impranil degradation ability among all the active fungi that were tested, based on the formation of a halo in medium plates. It is classified as part of the genus *Allophaeosphaeria* of the *Phaeosphaeriaceae* family. However, we found no references related to the degradation of *E. clematidis*. Thus, this is the first report on the ability of *E. clematidis* to degrade Impranil. Recently, *Phaeosphaeriaceae* was evaluated as a big family, consisting of more than 300 species of economically important plant pathogens, endophytes, or saprobes on plants (Phookamsak et al., 2014). The degradation activity suggests that the fungi in this family might be a promising source of biodiversity for testing important bioremediation activities.

We found that *E. clematidis* in solid and liquid screening assays (with Impranil as the sole carbon source) had the highest rate of Impranil degradation compared to other fungi in this study. This observation indicates that *E. clematidis* growth in the medium and its metabolism in the medium could be harnessed for Impranil degradation. The growth of *E. clematidis* was investigated through a modified Sturm test, which is based on the amount of CO_2_ released by the fungal culture in the presence and absence of Impranil. The growth of the fungus *E. clematidis* in the presence of ester hydrolysis products revealed that it utilized the products as a carbon source and mineralized to CO_2_ and H_2_O. Various studies used CO_2_ evolution tests to evaluate the degradation ability of microorganisms and found that several microbes released high amounts of CO_2_ during culturing, with polymer as a carbon source (Nakkabi et al., 2015; Bano et al., 2017; Hung et al., 2019). In the present study, the quantity of CO_2_ released by *E. clematidis* in the presence of Impranil as a carbon source was significantly higher than when Impranil is absent, suggesting that Impranil biodegradation was enhanced by fungal activity, as reflected by the corroborative levels of enzyme production and CO_2_ liberation.

Chemical composition analysis of the *E. clematidis* culture was performed using FTIR spectroscopy and GC-MS. Both techniques provide evidence to predict the mechanism of how the fungus attacks the polymer during biodegradation (Mahalakshmi et al., 2012; Shah et al., 2013; Muhonja et al., 2018). As far as the mechanism of degradation of Impranil is concerned, the fungus *E. clematidis* easily degraded the aliphatic segment to form various alcohol compounds detected by the GC-MS. However, the formulation of Impranil is proprietary (Biffinger et al., 2015). These compounds could be derived from both the polyester and polyisocyanate portions of PUR (Shah et al., 2013). FTIR analysis revealed that *E. clematidis* degraded the polyester portion of the Impranil. The dramatic variation of the carbonyl signal (1,715 cm^–1^) in the FTIR spectra were related to the attack of the ester bonds that are present in the polyol fractions, as well as the attack of the urethane groups (Mahalakshmi et al., 2012; Shah et al., 2013; Álvarez-Barragán et al., 2016; Muhonja et al., 2018). Russell et al. (2011) also reported that a decrease in the peak intensity at 1,715 cm^–1^ is consistent with the hydrolysis of the ester bond in the urethane linkage of PUR. Moreover, the decrease in the C-N-H bond peaks at 1,340 cm^–1^, and 1,020 cm^–1^ indicates that the urethane groups were eliminated. The reduction of the C=O and C-N-H signals confirms the degradation of Impranil by the fungus *E. clematidis*. The FTIR spectrum measured from the liquid medium without fungi showed a strong peak broadening at 3,270–3,335 cm^–1^, indicating the overlap of N-H and O-H- related peaks (Oprea, 2010). FTIR spectroscopy has been applied to monitor the variation in characteristic peaks related to polymer biodegradation.

Evidence of Impranil biodegradation by the fungus *E. clematidis* was also obtained by GC-MS analysis. New chemical compounds were detected after a 2-week incubation of *E. clematidis* and *A. niger* in the medium. The reduction of compounds containing ester bonds at the 15:00–38:00 min-mark increased alcohol compounds, probably due to the hydrolysis of the ester bonds in the polymer chain of PUR (Howard, 2002; Russell et al., 2011; Álvarez-Barragán et al., 2016). Ester compounds, 2E,4E-hexadienol acetate, and 3Z-hexenyl isobutanoate, may be synthesized by the combination of PUR precursors that are degraded by the fungi (Russell et al., 2011; Álvarez-Barragán et al., 2016). This work suggests that the use of fungi to degrade PUR waste presents an alternative, sustainable way of recovering PUR precursors for recycling. However, the compounds generated during the degradation of PUR by fungi might be related to oxidative reactions (Oprea, 2010; Yarahmadi et al., 2017).

FTIR spectroscopy and GC-MS were successfully applied for the analysis of chemical structure changes due to biodegradation by microorganisms. For example, Álvarez-Barragán et al. (2016) studied the biodegradative activities of selected environmental fungi on PUR. FTIR and GC-MS analyses revealed structural changes in PUR. A decrease in carbonyl groups (1,729 cm^–1^) and N-H bonds (1,540 and 1,261 cm^–1^) was measured by FTIR spectroscopy whereas a decrease in ester compounds and an increase in alcohols and hexane diisocyanate were shown by GC-MS analysis. The results obtained by both techniques indicated the hydrolysis of ester and urethane bonds. Shah et al. (2013) confirmed the degradation of PUR by *Pseudomonas aeruginosa* strain MZA-85 using FTIR and GC-MS. IR spectra indicated that the peak at 1,725 cm^–1^, which corresponds to ester linkage, decreased in the treated PUR samples, showing ester hydrolysis. The peak at 1,685 cm^–1^, obtained from the treated PUR sample, represents the overlap of carboxylic acid and amide carboxyl peaks, while the broadened N-H peak at 3,325 cm^–1^ represents the presence of free hydroxyl groups in the treated sample. The peak at 1,135 cm^–1^, which is attributed to the ester group was absent in the treated sample, confirming ester hydrolysis and the production of free hydroxyl groups after incubation with *P. aeruginosa* strain MZA-85. In addition, GC-MS analysis demonstrated two compounds, including 1,4-butanediol and adipic acid, which were detected in the treated sample only.

Many studies reported the esterase activities of microorganisms in the PUR degradation (Akutsu et al., 1998; Rowe and Howard, 2002; Mohanan et al., 2020). The enzymatic degradation of PUR occurs in two stages including adsorption of enzymes on the polymer surface in the first stage, followed by hydro-peroxidation/hydrolysis of the bonds in the second stage (Akutsu et al., 1998; Mohanan et al., 2020). Sato (1981) reported the protease, urease, and esterase activities produced by *A. niger* while culturing with PUR. Similarly, the structural changes due to Impranil degradation by the fungus *E. clematidis* are also correlated with the same enzymes. However, the rate of polymer biodegradation varied depending on several factors including chemical structures, and molecular weights (Mohanan et al., 2020). Of these three enzymes, esterase appears to be the most responsible for Impranil degradation. It is noted that the enzyme responsible for PUR degradation is extracellular, secreted, and diffusible (Oceguera-Cervantes et al., 2007; Loredo-Treviño et al., 2012; Hung et al., 2016). Extracellular enzymes secreted by microorganisms have played an important role in polymer degradation *via* depolymerization reaction by breaking down polymers into smaller molecules (Howard, 2002). The polymer chain is degraded by extracellular enzymes and becomes a degradation product. It can then be assimilated into microbial cells and used as carbon sources for producing carboxylic acid and alcohol that ultimately undergo respiration to produce energy (Kay et al., 1991; Nakajima-Kambe et al., 1995; Hung et al., 2016). In this study, extracellular enzymes esterase, lipase, and urease were investigated. *E. clematidis* was able to produce esterase enzymes which are believed to play a role in Impranil degradation. This result was in agreement with the study of Biffinger et al. (2015) showing the partial degradation of Impranil was significantly achieved by enzyme esterase. They reported that the enzymes (a *Pseudomonas* sp. esterase showed significant esterase activities and partially cleared Impranil by partial hydrolysis. Esterase enzymes have been isolated and reported by several studies to degrade PUR. Noor et al. (2020) found esterase in the outer membrane of *P. aeruginosa*, demonstrating a particular affinity to long-chain acyl esters. Purified esterase was also isolated from the outer membrane of PUR-degrading bacteria *C. acidovorans* strain TB35 and *P. aeruginosa* PAO1 (Akutsu et al., 1998; Wilhelm et al., 1999). Mukherjee et al. (2011) also reported the ability of esterase enzymes from *P. aeruginosa* to degrade polyurethane diol. Moreover, esterase was purified from a PUR-degrading fungus, *Curvularia senegalensis* (Loredo-Treviño et al., 2012). The esterase enzyme attacked the ester bond cleavage in the soft segments of PUR. Yu et al. (2010) reported the role of esterase in the catalysis of the cleavage of ester bonds in the polymer chain in PUR. In addition, 6-hydroxyhexanoate was generated from the hydrolysis of the ester linkages in polycaprolactone polyol-based PUR by an esterase enzyme (Magnin et al., 2019). Shah et al. (2013) also described that esterase that was purified from the strain *P. aeruginosa* MZA-85 specifically damaged the ester bonds in the aliphatic or soft segment, leading to the depolymerization of the polymer chain to degradation products. These products could be further metabolized through different metabolic pathways (Brzostowicz et al., 2002; Yoshida et al., 2016). However, there is limited information about the mechanism of degradation of Impranil by esterase secreted from *E. clematidis*. Therefore, the mechanism of how esterase is secreted by *E. clematidis* should be further studied in detail. Optimal conditions for the fungal activity must also be improved before applying it on a larger scale for commercial use.

## Conclusion

This work establishes that various fungi are useful biodiversity sources with potential application in Impranil degradation. It is interesting to note that *E. clematidis* had the highest capacity for Impranil degrading. FTIR spectroscopy and GC-MS analysis indicate that Impranil was successfully degraded by *E. clematidis*, with Impranil as the sole carbon source. The fungus *E. clematidis* degraded the polymer chain of Impranil, constituting the soft segment, and produced degradation products such as 5Z-octenol, and 2E,4E-hexadienol acetate. The ester and urethane groups in the polymer chain of Impranil were attacked *via* the esterase enzyme. Further studies, which explain the mechanism behind the esterase enzyme production of *E. clematidis* and its role in PUR degradation, are required for its potential application in polyurethane waste management.

## Data Availability Statement

The raw data supporting the conclusions of this article will be made available by the authors, without undue reservation.

## Author Contributions

SK and TS performed experiments on solid- and liquid-medium assays. SK performed FTIR, GC-MS analysis, Sturm test, enzyme activity assay, analyzed data, and wrote the first draft of the manuscript. PP edited, reviewed the manuscript, and supervised the work. All authors contributed to the article and approved the submitted version.

## Conflict of Interest

The authors declare that the research was conducted in the absence of any commercial or financial relationships that could be construed as a potential conflict of interest.

## Publisher’s Note

All claims expressed in this article are solely those of the authors and do not necessarily represent those of their affiliated organizations, or those of the publisher, the editors and the reviewers. Any product that may be evaluated in this article, or claim that may be made by its manufacturer, is not guaranteed or endorsed by the publisher.
